# Assessing Sarcocornia as a Salt Substitute: Effects on Lipid Profile and Gelatinase Activity

**DOI:** 10.3390/nu16070929

**Published:** 2024-03-23

**Authors:** Beatriz Louçano, Sara Maletti, Helena Timóteo, João Paulo Figueiredo, Nádia Osório, Maria João Barroca, Aida Moreira da Silva, Telmo Pereira, Armando Caseiro

**Affiliations:** 1 Polytechnic Institute of Coimbra, Coimbra Health School, Biomedical Laboratory Sciences, Rua 5 de Outubro, S. Martinho do Bispo, 3046-854 Coimbra, Portugal; beatrizloucano@hotmail.com (B.L.); mariatimoteo5@gmail.com (H.T.); nadia.osorio@estesc.ipc.pt (N.O.); armandocaseiro@estesc.ipc.pt (A.C.); 2 Surgical, Medical and Dental Department of Morphological Sciences Related to Transplant, Oncology and Regenerative Medicine, Faculty of Medicine and Surgery, University of Modena and Reggio Emilia, Policlinico, via del Pozzo, 7141124 Modena, Italy; 301732@studenti.unimore.it; 3 Polytechnic Institute of Coimbra, Coimbra Health School, Medical Sciences, Socials and Humans, Rua 5 de Outubro, 3046-854 Coimbra, Portugal; jpfigueiredo@estesc.ipc.pt; 4 LABINSAÚDE-Research Laboratory for Applied Health Sciences, Polytechnic Institute of Coimbra, Coimbra Health School, Rua 5 de Outubro, S. Martinho do Bispo, 3046-854 Coimbra, Portugal; telmo@estesc.ipc.pt; 5 Molecular Physical-Chemistry R&D Unit, Department of Chemistry, University of Coimbra, 3004-535 Coimbra, Portugal; aidams@esac.pt; 6 Polytechnic Institute of Coimbra, Agriculture School of Coimbra, Bencanta, 3040-360 Coimbra, Portugal; 7 Polytechnic Institute of Coimbra, Coimbra Health School, Clinical Physiology, Rua 5 de Outubro, S. Martinho do Bispo, 3046-854 Coimbra, Portugal; 8 Faculty of Sport Science and Physical Education, University of Coimbra, CIDAF—Research Unit for Sport and Physical Activity, 3000-456 Coimbra, Portugal

**Keywords:** salt-tolerant plants, sodium chloride, matrix metalloproteinase 2, matrix metalloproteinase 9, cholesterol, *Sarcocornia perennis*

## Abstract

Sodium, although essential for life, is a key factor in changes in vascular function and cardiovascular disease when consumed in excess. *Sarcocornia* spp., a halophyte plant with many nutritional benefits, presents itself as a promising substitute for the consumption of purified salt. Matrix metalloproteinases (MMPs) 2 and 9 are widely studied due to their action in physiological processes and as biomarkers at the diagnostic level due to their increased expression in inflammatory processes. This study aimed to evaluate whether replacing salt with *Sarcocornia perennis* (*S. perennis*) powder in healthy young people leads to an improvement in biochemical profiles and the attenuation of MMP-2 and MMP-9 activity. In the present study, 30 participants were randomized into a control group that consumed salt and an intervention group that replaced salt with powdered *S. perennis*. The evaluation of the biochemical parameters was carried out by the spectrophotometry method, and the evaluation of MMP activity was carried out by zymography. A significant decrease was observed in the intervention group in total cholesterol, high-density lipoprotein cholesterol (HDL-c), and creatinine (*p*-value ≤ 0.05), along with lower but not significantly different mean values of triglycerides. Regarding MMP activity after the intervention, a lower mean value was observed for MMP-9 activity, with there being higher mean values for MMP-2 activity, both with *p*-values ≥ 0.05. The results confirmed that the consumption of *S. perennis* is a beneficial choice for health regarding the lipid profile. The evaluation of MMP activity indicated the potential of *S. perennis* in the regulation of MMP-9 activity in healthy individuals, along with the need for the further study of these proteases in individuals with pathologies.

## 1. Introduction

Sodium is an extremely abundant nutrient, essential for life and the good functioning of organisms. It plays a role in diverse physiological processes like body fluid homeostasis, blood pressure (BP) regulation, metabolic functions, and kidney function and, at a muscular and neuronal level, is indispensable for maintaining the balance between the levels of consumption and excretion [1,2,3,4].

The World Health Organization (WHO) affirms that one’s daily consumption of sodium should not exceed 2 g (5 g of salt) due to the direct relation between the excessive consumption of sodium and increased BP levels, although an individual’s daily sodium consumption, as described in several studies, is estimated to be around 9–12 g of salt worldwide [3,4,5,6,7,8,9,10,11,12,13]. According to Alfheeaid et al. [12], about 99.2% of the adult world population presents levels of sodium consumption higher than those recommended by the WHO, with this being related to the poor nutrition in the Western diet [14].

Excess sodium consumption is clearly highlighted in the literature as being responsible for triggering an increase in water retention, blood flow pressure, inflammatory processes, progressive arterial stiffness, high BP levels, cardiovascular diseases (CDs), and changes in the intestinal microbiome, meaning it is a problem worldwide [1,2,3,5,12,15].

With this in mind, it is necessary to reduce salt intake or replace salt with healthy alternatives, as increasing studies suggest that this is a promising preventive strategy, especially in hypertensive patients [3,5,6,16,17,18].

The opportunity to study halophyte plants emerged; these are among the plants with the best salt tolerance in the world, presenting several cultivation, economic, and nutritional advantages [9,19,20,21,22,23,24]. They own adaptative mechanisms that allow them to grow worldwide in environments with high salinity, being dispersed in coastal zones; subtropical, subarctic, and brackish areas; and saline deserts [9,12,14,19,20,21,22,23]. 

Within halophyte plants, two very important genera are *Sarcocornia* spp. and *Salicornia* spp., belonging to the family *Amaranthaceae* and subfamily *Salicornioideae* [12,14,25]. According to the literature, both present about 30 species each, and they are very similar morphologically, ecologically, and taxonomically [9,14]. 

These plants are also known as glassworts, samphires, pickleweeds, and sea asparagus and have a salty flavor, crunchy texture, and slight fibrousness [9,14,21,23]. Other common synonyms of *Sarcocornia perennis* (*S. perennis*) are *Salicornia perennis* Mill., and *Arthrocnemum perenne* (Mill.) Fourc. [26,27].

It is possible to find the introduction of some halophytes in the form of gourmet foods such as garnishes or side dishes. They can be consumed fresh, boiled, powdered, conserved/preserved, fermented, or dehydrated, and throughout history, halophytes have been used in traditional medicine to treat gastrointestinal problems, inflammation, diabetes, and hypertension [3,9,12,20,21,22,23]. They present an advantageous nutritional profile and antioxidant, anti-inflammatory, anticancer, antibacterial, and antihypertensive properties [9,12,14,20,21,22,23]. Also, studies show that given their health benefits and salty flavor, halophytes are a promising option for replacing the consumption of purified salt [3,9,19,20,21,22]. 

Matrix metalloproteinases (MMPs) are proteolytic enzymes that are dependent on zinc and calcium and synthesized in conjunctive tissues, endothelial cells, vascular smooth muscle, and pro-inflammatory cells. There are at least 28 types of MMPs in vertebrates, 24 in humans [28,29,30,31,32,33]. They present structural similarities, but in an inactive form, they are called zymogens or proMMPs [28,29,31,32,33]. These enzymes are classified according to the organization of their structural specificities and their substrates, with an emphasis on the following types: collagenases, gelatinases, stromelysins, and matrilysins of the membrane type, among others [28,29,31,32,33].

MMPs are found in all layers of the vascular walls, and their functions go through several physiological processes, like tissue remodeling, vascular remodeling through the degradation of collagen and elastin, embryogenesis, morphogenesis, healing, bone remodeling, the activation of immune cells, cell migration, and proliferation [28,29,30,31,32,33,34]. 

The regulation of MMPs is carried out through several steps and by the action of tissue inhibitors of metalloproteins that reversibly block the action of MMPs [31,32,34].

Due to an imbalance between the expression of MMPs and their inhibitors, there is an overexpression of MMPs in various tissues, which leads to the development of inflammatory conditions, cell proliferation, the excessive degradation of matrix components, autoimmune diseases, carcinogenesis, fibrosis, arterial stiffness, CD, and lung and neurological diseases [29,30,31,32,33,34]. Studies affirm that determining the activity of theses enzymes is indeed a promising key factor given the possibilities of better diagnosis, prognosis, and the monitoring of various diseases [31,32,33].

Gelatinase-type MMPs like MMP-2 and MMP-9 display physiological functions such as remodeling the extracellular matrix (ECM), but in pathological situations, they end up degrading gelatin, elastin, and type IV collagen in excess, as referenced in several studies [3,27,28,29,31,33].

The present study aimed to evaluate the impact of replacing salt consumption with the use of dried powder (*S. perennis*) for 30 days through a randomized clinical trial. The intervention was evaluated by determining the participants’ biochemical profiles and the activity of MMP-2 and MMP-9.

## 2. Materials and Methods

### 2.1. Halophyte Plant

*S. perennis* was provided by the Salina Greens company (Alcochete, Setúbal, Portugal). The nutritional and mineral composition of dried *S. perennis* (powder) can be found elsewhere in [24]. This powder boasts significant levels of proteins (16.7 g/100 g) and carbohydrates (39.5 g/100 g), alongside a total ash content of 33.9 g/100 g, indicative of its rich mineral profile.

Among the prominent minerals present in *S. perennis* powder are sodium (7119.35 mg/100 g), potassium (1830.54 mg/100 g), magnesium (786.39 mg/100 g), calcium (490.87 mg/100 g), and traces of phosphorus, iron, zinc, and manganese. Furthermore, S. perennis extract has a high content of phenolic compounds (15.79 mg GAE/g) and antioxidant capacity (58.49 mg Trolox/g), as measured by the DPPH method [24].

### 2.2. Study Sample

The study sample included 30 participants, all young people aged over 18 years old (medium de 20.4 ± 1.2), with 23.3% being male and 76.7% female (Table 1) [3]. This research project was approved by the Ethics Committee of the Polytechnic Institute of Coimbra (7/2019, approved on 18 September 2019) and conducted in accordance with all the principles of the Declaration of Helsinki [3].

The participants were randomly divided into two groups equally. The control group (CG), with 15 participants (*n* = 15), maintained the consumption of added salt in their diet, and those in the intervention group (IG), also with 15 participants (*n* = 15), were instructed to replace their salt intake with *S. perennis* powder, previously studied, and use it for 30 days in the desired amount in order to obtain a salty flavor, as mentioned by Pereira et al. [3]. The inclusion criteria encompassed individuals aged over 18 years without a diagnosis of hypertension or associated comorbidities. The exclusion criteria applied were allergic processes, diagnosis of secondary hypertension, and hypertensive patients undergoing medication.

Vascular physiology of the population was characterized before and after the intervention via assessments of blood pressure and pulse wave velocity (PWV) [3]. Sodium excretion was quantified using spot urine samples stored at −70 °C. The Tanaka method was performed as described by Iida et al. [35], which, through the quantification of sodium and creatinine levels, allowed us to estimate saline excretion in 24 h [3].

After carrying out the characterization of vascular physiology according to Pereira et al. [3], we confirmed a significant decrease in BP values and improvement in PWV in the IG. Concerning the amount of sodium excreted in 24 h, as described by Pereira et al. [3], the CG presented an average value of 8.4 ± 1.8 g/day before the intervention and 8.5 ± 2.4 g/day after the intervention, while the IG presented an average value of sodium excretion of 8.9 ± 2.1 g/day before the intervention, and after the consumption of *S. perennis*, the medium value became 7.2 ± 1.2 g/day, denoting a reduction in sodium excretion within the IG.

### 2.3. Instruments and Data Collection

The collection of data and biological samples were carried out at two different moments of the study: at the time of the first contact with the participants and collection of clinical data (T0) and 30 days after the intervention (T1).

All data obtained were recorded, organized, and subsequently subjected to statistical analysis using the IBM SPSS Statistics 28^®^ program (Armonk, NY, USA). At the same time, blood samples were taken [3]. For the biochemical profiles, blood samples were taken at moments T0 and T1 to compare between the two collection moments. Venous blood samples were collected into 10 mL dry gel tubes to obtain the participants’ serum. These tubes were posteriorly centrifuged at 3500× *g* for 10 min, and the serum samples were conditioned and stored at a temperature of −70 °C until further analysis [36,37].

#### 2.3.1. Characterization of the Biochemical Profiles

To evaluate the biochemical profiles, we used Prestige 24i equipment (Tokyo Boeki, Tokyo, Japan) based on the spectrophotometry method and using Cormay Prestige 24i kits (PZ Cormay S.A., Warsaw, Poland). The following parameters were evaluated: glucose, total cholesterol, high-density lipoprotein cholesterol (HDL-c), triglycerides, creatinine, aspartate aminotransferase (ASAT), and alanine aminotransferase (ALAT) [36,37,38].

#### 2.3.2. Evaluation of the Activity of Matrix Metalloproteinases 2 and 9 

The determination of MMP-2 and MMP-9 activity in serum was carried out using the zymography technique as described by Vitorino et al. [39,40,41]. This technique is based on protein separation using the SDS-PAGE method (sodium dodecyl–sulfate polyacrylamide gel electrophoresis) and is a useful tool for determining MMP activity in several biological samples, such as serum, urine, or saliva [36,39,40,42].

After protein quantitation, 10 µg of each serum sample was loaded into the stacking gel [36,41,43]. Two molecular weight standards were used, a commercial one (Precision Plus Protein Kaleidoscope Standards, BIO-RAD, California, EUA) and a capillary blood standard. The gels were previously prepared, with the running gel having a concentration of 10% and 0.1% of gelatin, while the stacking gel had a concentration of 4% [36,39,41,44,45].

The proteins were separated by using the SDS-PAGE technique for 1 h at 180 volts [36,39,40,42]. After electrophoresis, the gels were incubated twice in the renaturation solution, 2.5% Triton X-100, for 30 min at room temperature and under agitation [36,39,40,42].

Then, the gels were placed in development buffer, pH 7.4, and after initial incubation at room temperature and under agitation, the gels were incubated overnight (16 h) at around 37 °C in new development buffer [36,39,40,42].

Finally, the gels were stained with Coomassie Brilliant Blue G250 0.5% *w/v* and destained with 40% methanol/10% acetic acid. We obtained bright bands against a dark blue background representing the undegraded substrate [39,41,42]. Images of the gels were taken using the GelDoc XR system (Bio-Rad, Hercules, CA, USA) and analyzed with ImageLabR version 3.0 software (Bio-Rad Hercules, USA) [36,37,39,41]. 

### 2.4. Statistical Analysis

To collect data and interpret the results, the Microsoft Excel program was used, and the results were subsequently transferred to the IBM SPSS Statistics 28^®^ software (Armonk, NY, USA), which was used to carry out statistical analysis. The normality of the data and the assumption of asymmetry and flattening were assessed using the Shapiro–Wilk test (*n* ≤ 50), Skewness Coefficient, and Kurtosis Coefficient. Student’s parametric *t*-test for paired samples was also used with the aim of observing the changes in each group (intervention and control groups). The difference between the groups under study were assessed as statistically significant when the random error *p*-value ≤ 0.05 with a confidence level of 95% or higher. The determination of MMP activity was expressed in arbitrary units (AU).

## 3. Results

### 3.1. Biochemical Evaluation

As previously described, the biochemical evaluation of our participants was carried out at T0 and T1 in order to guarantee comparable conditions at both time points/moments using Student’s parametric *t*-test. 

In this way, it was possible to interpret the results and compare the CG and IG, and after interpreting the biochemical parameters results, it was observed that there were no statistically significant differences between the groups in T0 (*p*-value ≥ 0.05) (Table 2). 

**Figure 1:**
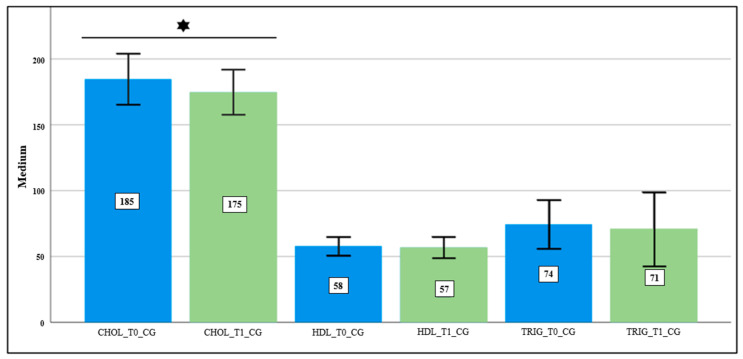
Evaluation of lipid profiles in CG at T0 and T1; CHOL_T0_CG: total cholesterol at T0 of CG; CHOL_T1_CG: total cholesterol at T1 of CG; HDL_T0_CG: HDL-c at T0 of CG; HDL_T0_CG: HDL-c at T1 of CG; TRIG_T0_CG: triglycerides at T0 of CG; TRIG_T1_CG: triglycerides at T1 of CG; confidence level of 95%; 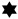
—Student’s *t*-test was performed, which was statistically significant; *p*-value ≤ 0.05.

**Figure 2:**
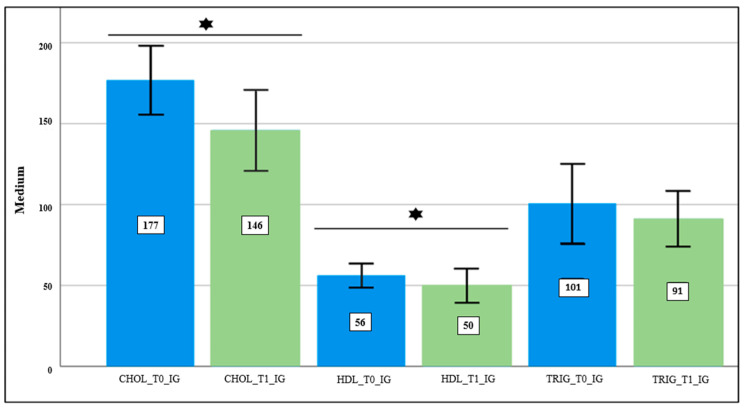
Evaluation of lipid profiles in IG at T0 and T1; CHOL_T0_IG: total cholesterol at T0 of IG; CHOL_T1_IG: total cholesterol at T1 of IG; HDL_T0_IG: HDL-c at T0 of IG; HDL_T0_IG: HDL-c at T1 of IG; TRIG_T0_IG: triglycerides at T0 of IG; TRIG_T1_IG: triglycerides at T1 of IG; confidence level of 95%; 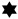
—Student’s *t*-test was performed, which was statistically significant; *p*-value ≤ 0.05.

The interpretation of the biochemical parameters results in the CG only showed lower mean values at T1 in total cholesterol (*p*-value ≤ 0.05) (Figure 1). With regard to the evaluation of the biochemical parameters in the IG, a statistically significant decrease was observed between moments T0 and T1 in total cholesterol, HDL-c, and creatinine (Figure 2). Regarding glucose, ALAT, ASAT, and triglycerides, we found a tendency for the mean values to decrease after the experimental period, although this trend was not statistically significant (Table 3 and Figure 2).

### 3.2. Evaluation of the MMPs’ Activity

When carrying out the zymography technique to determine the MMP-9 and MMP-2 activity, two dropouts occurred because insufficient samples, meaning our final sample consisted of 28 participants. 

**Figure 3:**
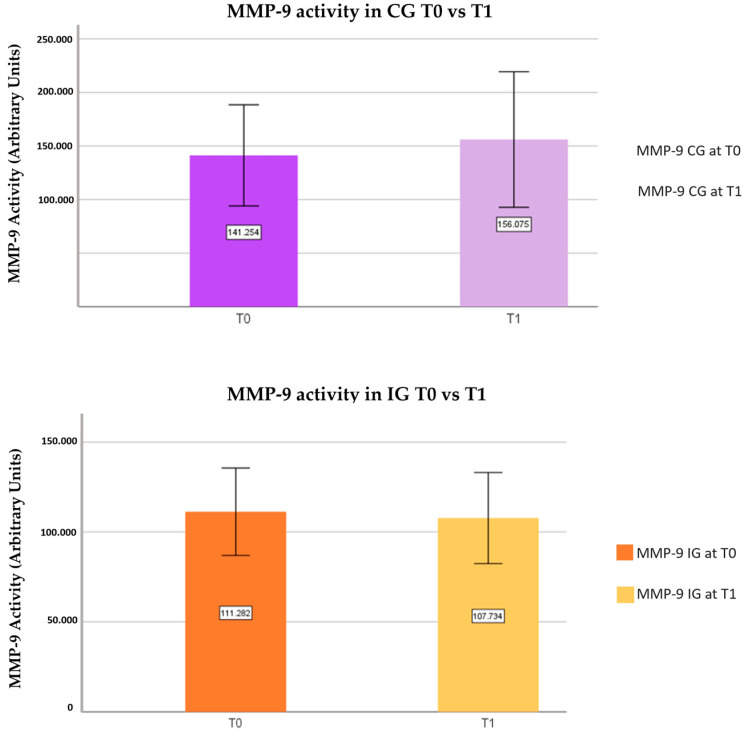
Evaluation of MMP-9 activity in the CG and IG at moments T0 and T1.

**Figure 4:**
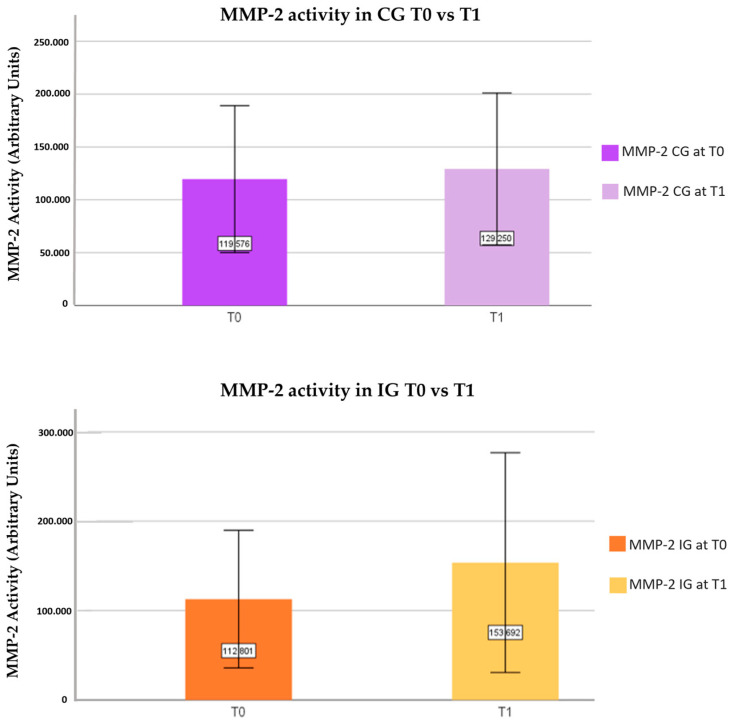
Evaluation of MMP-2 activity in the CG and IG at moments T0 and T1.

In the CG, the average value of MMP-9 activity at T1 was 156,075 ± 109,537 compared to 141,254 ± 81,765 at T0, with no statistically significant differences being observed (Figure 3). Regarding MMP-2 in the CG, the mean activity value at T1 was 129,250 ± 124,465 compared to 119,576 ± 120,439 at T0, also without statistically significant differences being observed (Figure 4).

Concerning MMP-9 in IG, the mean value of MMP-9 activity observed at T1 was 107,734 ± 43,859 compared to 111,282 ± 42,120 at T0, presenting a slight decrease in the activity at T1 compared to T0, although not statistically significant (Figure 3). Regarding MMP-2, the average activity value observed at T1 was 153,692 ± 213,080 compared to 112,801 ± 133,463 at T0. A trend involving an increase in the activity at T1 was observed, with no statistical significance (Figure 4).

## 4. Discussion

This randomized study aimed to characterize a population of clinically healthy young people through an analysis of their biochemical profiles, as well MMP-2 and MMP-9 activity, to demonstrate the possible benefits of replacing salt with dried powder of *S. perennis*.

The intake of high amounts of salt and its relationship with the development of various pathologies has been widely studied in humans and animals [15,19,46]. 

Halophyte plants present themselves as excellent sources of bioactive compounds such as flavonoids, phenolic compounds, carotenoids, saponins, tannins, and minerals. Flavonoids are a diverse group of phytonutrients known for their antioxidant properties. Flavonoids found in *Sarcocornia* plants may contribute to their ability to scavenge free radicals and protect against oxidative stress. Phenolic compounds are another group of antioxidants present in many plant species, including *Sarcocornia*. These compounds have been associated with various health benefits, including anti-inflammatory and cardioprotective effects. Carotenoids are pigments responsible for the vibrant colors of many fruits and vegetables. They also act as antioxidants and may have protective effects against certain chronic diseases, such as cardiovascular disease and age-related macular degeneration. Saponins are naturally occurring compounds with diverse biological activities, including antimicrobial, anti-inflammatory, and immunomodulatory properties. They are often found in plants and may contribute to the medicinal properties of *Sarcocornia* species. Tannins are polyphenolic compounds that can bind to and precipitate proteins. They have been studied for their antioxidant and anti-inflammatory effects and may contribute to the medicinal properties of *Sarcocornia* plants. *Sarcocornia* plants, being halophytes, have adapted to grow in saline environments and can accumulate high levels of minerals such as sodium, potassium, magnesium, and calcium. These minerals are essential for various physiological functions in the human body [47]. Bioactive compounds act in a preventive and mitigating manner in various pathologies thanks to their antioxidant, anti-inflammatory, anti-diabetic, anticancer, antibacterial, antihypertensive, neuro-protective, and anti-dyslipidemic properties, but they can also act as promising salt substitutes, thus enabling their use in the food and pharmaceutical industries [9,12,14,15,19,20,21,22,23,47,48,49,50,51,52,53]. 

Previous studies have shown that *Salicornia* provides a protective effect at the vascular level, preventing endothelial dysfunction, hypertension, and CD, with its action being referenced in several studies, such as that by Lopes et al. [11], which revealed improvements in the vascular system following the administration of comparable amounts of salt and *Salicornia* in rats [12,19,22,53,54]. In the present study, it was also possible to highlight an improvement in vascular physiology in the IG compared to the CG, which was confirmed by Pereira et al. (2023) [3]. Also, D’Elia et al. states that a diet with reduced salt intake results in an improvement in PWV [15,55].

Several studies highlight the nutritional value of the genus *Salicornia* spp. due to the presence of phenolic acids such as caffeic acid, ferulic acid, and p-coumaric acid; flavonoids such as isoquercitrin, quercetin, luteolin, and kaempferil; high-quality fatty acids such as oleic acid, linoleic acid, and palmitic acid; amino acids such as arginine, aspartic acid, leucine, glutamic acid, and isoleucine; vitamins A, B, C, and D; β-carotenes; proteins; carbohydrates; and numerous mineral salts, such as Na, Mg, K, Ca, and Fe [9,12,14,19,20,21,22,23].

Concerning the evaluation of biochemical parameters, significant results were only observed in the CG regarding total cholesterol, with a slight decrease in mean values at T1 (Figure 1). Significant differences were also observed in the IG group at T1 compared to at T0 in the parameters of total cholesterol, HDL-c, and creatinine (Table 3 and Figure 2). There was also a tendency towards a decrease in triglycerides values, which did not show significant differences (Figure 2). Despite our sample comprising clinically healthy young individuals, extant research indicates a favorable association between halophyte consumption and enhancements in lipid profiles.

In this context, Lee Ji Hwan et al. highlighted that the administration of *Salicornia* extract in db/db rats allows for an improvement in obesity by inhibiting adipocyte differentiation. Additionally, a concomitant decrease in LDL-c and triglyceride levels was observed, supporting the idea that there is an improvement in metabolism and dyslipidemia [56].

Another study, specifically that carried out by Rahman et al. [57], presented a consensus with previous studies, as the anti-obesity effect of *Salicornia* supplementation in Sprague Dawley (SD) rats resulted in a reduction in body mass and abdominal measurements and improvements in lipid profiles. According to Zhang et al. [54], a tendency towards a decrease in serum creatinine values was observed in SD rats following *Salicornia* consumption, as well as protective effects on the liver, kidney, and a decrease in BP level. In line with these findings, a statistically significant decrease in creatinine levels was observed within the IG at T1 compared to T0 (Table 3) [12].

To reinforce, DaeKeun et al. found that the use of Salicornia as a dietary supplement in SD rats resulted in a tendency towards a decrease in triglycerides, although body mass remained the same [58]. Chrigui et al. [25], through a study carried out on dyslipidemic and obese *Psammomys obesus*, highlighted that after the administration of *Salicornia* extract, there was a decrease in BMI, a decrease in the accumulation of adipocytes in the liver, and a decrease in weight, thus confirming anti-dyslipidemia effects. 

In the present study, lower mean values of ASAT and ALAT were also observed in the IG at T1 compared to T0 (Table 3), without statistically significant differences, which is in line with what was reported by Chrigui et al. [25], leading us to believe there was a possible preventive effect on liver tissue damage after the consumption of *Salicornia*.

Of the minerals present in *S. perennis*, it is worth highlighting potassium and magnesium, which favor saline excretion, allowing for the harmful effects of salt in the body to be reduced, as described by Pereira et al. [3]. 

Vitamins (A, C, D, β-carotenes) act as a source of natural antioxidants, improving endothelial dysfunction, preventing CD and degenerative diseases, reducing inflammatory processes, and eradicating free radicals in order to attenuate oxidative stress [3,14,15,19,59,60,61,62,63,64,65,66,67,68]. In this context, Ulker et al. reported a positive relationship with vascular function in hypertensive rats after the administration of vitamin C [67,68]. 

Given the components present in *Salicornia* and other similar halophytes, Kong et al. highlights the flavonoid 3-O-β-D-glucopyranoside as a promising anti-obesity treatment, and other studies report that flavonoids also have antioxidant and anti-inflammatory properties [15,19,59,69,70]. The consumption of unsaturated fatty acids such as oleic acid and linoleic acid, present in *Salicornia*, reduces the risk of developing CD and leads to benefits in terms of the lipid profile [12,15,19,71,72,73,74,75].

Given the nutritional profile of *S. perennis*, a tendency towards a decrease in the biochemical parameters of the IG was observed, which agrees with the studies referenced previously. On the other hand, studies have reported improvements in animals and humans at the metabolic level after consuming halophytes based on pathological samples. Therefore, we can assume that, given our results present significant differences after the intervention in a healthy population, the same should be observed in a sick population, possibly even with a greater magnitude. 

As mentioned in the literature, MMPs are expressed in different tissues and participate in different physiological processes [28,30,31]. Any deregulation in their activity promotes the excessive degradation of the ECM, chronic inflammation, and oxidative stress, promoting various diseases [30,31]. However, MMPs can be activated in different ways, with oxidative stress leading to a high production of reactive oxygen species and, consequently, the activation of MMPs, causing a progressive increase in vasoconstriction [30,31]. It has also been described that antioxidants can inhibit the activation of MMPs, leading to improvements in vascular function [30]. Therefore, given the antioxidant and anti-inflammatory benefits of *S. perennis*, it may have scientific relevance in terms of MMP activation. 

The activity of MMP-2 and MMP-9 has been widely studied in animals and humans, presenting itself as a promising diagnostic biomarker and therapeutic target in various pathologies, such as changes in the vascular system, ischemic lesions, neurodegenerative diseases, lung diseases, and cancer [28,39,76]. MMP-2 is constitutively present in the vessel wall, and MMP-9 is widely associated with inflammatory processes [28].

The MMP activity results in this study were interpreted based on the observation of clear bands (degraded substrate) in contrast to the blue-colored gel. The commercial and capillary standards allowed us to identify the respective bands corresponding to the molecular weights of the MMPs under study [39,40,42]. MMP-2 has a molecular weight of around 72 kDa in the zymogen form and 64 kDa in the active form, while MMP-9 has a molecular weight of around 92 kDa for the zymogen from and 86 kDa for the active form [39,40,42].

In the present study, no significant differences were observed in the assessment of MMP-2 and MMP-9 activity in the CG or IG. Slightly lower mean values of MMP-9 activity were observed in the IG at T1 compared to T0, with no significant differences (Figure 3). We verified slightly higher mean values of MMP-2 and MMP-9 activity from T0 to T1 in the CG (Figure 3 and Figure 4), as well as a slight increase in the mean values of MMP-2 activity at T1 in the IG (Figure 4). Contrary to the results obtained, although the sample in the present study is clinically healthy, Valente et al. evaluated the expression of MMP-9 in groups with hypertensive crises, controlled hypertension, and normotensives, and statistically significant results were obtained that proved that the group in with emerging hypertensive crises presented higher levels of MMP-9 compared to the normotensive group, supporting that MMP-9 levels are related to inflammatory processes and an increased risk of CD [77]. However, no differences in MMP-9 levels were identified between the normotensive group and the controlled hypertensive group, possibly due to the use of antihypertensive medication [77,78]. Furthermore, it was mentioned that the identification of high levels of MMP-9 in healthy people may prove to be a predictive marker for the risk of developing CD [77,79]. 

In another study, evaluating MMP levels in obesity revealed that in obese individuals, the level of MMP-2 is higher compared to overweight or non-obese individuals [80]. However, MMP-9 levels were not concrete, with reduced levels of MMP-9 being detected in obese individuals, as well as several discrepancies in results compared to those of other studies, leading to the belief that they are dependent on several factors, such as gender, age, and technique(s) used [80].

On the other hand, Campino et al. evaluated the expression of MMP-2 and MMP-9 in two groups in order to understand whether salt consumption was associated with endothelial damage and metabolic dysregulation. Finally, no significant differences were observed in the expression of MMP-2 and MMP-9, although it was possible to verify a tendency towards a decrease in both MMPs in the group that consumed adequate salt compared to the group that consumed salt in high quantities [81]. MMP-2 is presented as a ubiquitous protease, being associated with pro-inflammatory and anti-inflammatory situations [82,83]. D‘Avila-Mesquita et al. reported that in COVID-19 patients with a severe inflammatory state, MMP-2 activity was reduced, in agreement with previous studies which report reduced levels of MMP-2 in patients with clinical signs of sepsis [84,85]. Cancemi et al. also revealed that high levels of MMP-2 activity may suggest greater longevity [82]. 

This study has limitations such as its sample size (*n* = 30) and the difficulty in controlling the amounts of salt and *S. perennis* ingested by our participants, although consumption instructions were given [3].

According to what has previously been mentioned, even though our study was based on a clinically healthy sample, we were able to verify improvements in lipid profiles; possibly, a larger sample could confirm more confidently the trends observed regarding both biochemical profiles and MMP activity.

For future studies, it will be important to continue research into the use of *S. perennis*, as this is necessary to ensure that its benefits for human health are well reported, allowing for it to be used on a regular and expanded basis in diverse applications, such as supplementation, salt replacement, or in the pharmaceutical industry. 

## 5. Conclusions

The consumption of *S. perennis* by healthy young people results in an improvement in lipid profiles, as well as a tendency towards a decrease in MMP-9 activity.

It will be pertinent to evaluate MMP-2 activity in relation to the consumption of *S. perennis* in more detail and encourage research with a greater number of participants or even unhealthy participants so that the numerous advantages that this halophyte plant provides to human health as a salt substitute can be confirmed with greater confidence.

## Figures and Tables

**Table 1:** Characterization of the population in relation to qualitative variables.

	CG (*n* = 15)	IG (*n* = 15)	Total (*n* = 30)	P
Age	20.6 ± 1.5	20.2 ± 0.9	20.4 ± 1.2	0.379
BMI	22.8 ± 1.9	21.3 ± 2.7	22.1 ± 2.4	0.085
Waist	74.6 ± 8.0	77.8 ± 8.4	76.2 ± 8.1	0.296
Hip	94.4 ± 8.4	94.8 ± 7.9	94.6 ± 7.9	0.894
Ad. MD	7.3 ± 1.4	6.6 ± 1.7	7.0 ± 1.5	0.205

Legend: CG—control group; IG—intervention group; BMI: body mass index; Ad. MD: adherence to the Mediterranean diet; P: *p*-value.

**Table 2:** Characterization of the population based on biochemical parameters.

	CG (*n* = 15)	IG (*n* = 15)	P
Total Cholesterol *	183.5 ± 32.6	176.9 ± 35.8	0.372
HDL-c *	59.8 ± 13.8	56.1 ± 12.5	0.206
Glucose *	84.0 ± 9.2	82.5 ± 5.2	0.419
Creatinine *	0.8 ± 0.1	0.8 ± 0.1	0.372
ALAT	17.2 ± 10.0	18.3 ± 8.7	0.372
ASAT	21.7 ± 4.7	20.6 ± 5.4	0.218
Triglycerides *	74.3 ± 31.1	100.7 ± 42.4	0.368

Legend: CG–control group; IG–intervention group; HDL-c: cholesterol associated with high-density lipoproteins; ALAT: alanine aminotransferase; ASAT: aspartate aminotransferase; P: *p*-value. * units of measurement—mg/dL; ALAT and ASAT—units of measurement U/L.

**Table 3:** Evaluation of biochemical parameters in the control group (CG) and intervention group (IG).

	CG (*n* = 15)			IG (*n* = 15)		
	T0	T1	P	T0	T1	P
Total Cholesterol *	183.5 ± 32.6	173.0 ± 29.4	0.008	176.9 ± 35.8	149.7 ± 43.8	0.031
Glucose *	84.0 ± 9.2	83.7 ± 7.3	0.314	82.5 ± 5.2	80.1 ± 14.8	0.128
Creatinine *	0.8 ± 0.1	0.8 ± 0.1	0.275	0.8 ± 0.1	0.7 ± 0.1	0.014
ALAT	17.2 ± 10.0	15.3 ± 4.5	0.324	18.3 ± 8.7	16.2 ± 8.4	0.426
ASAT	21.7 ± 4.7	20.7 ± 2.8	0.239	20.6 ± 5.4	18.6 ± 5.8	0.091

Legend: CG—control group; IG—intervention group T0—first contact with the participants; T1—moment one month after the intervention; ALAT: alanine aminotransferase; ASAT: aspartate aminotransferase; P: *p*-value; * units of measurement—mg/dL; ALAT and ASAT—units of measurement U/L.

## Data Availability

The original contributions presented in the study are included in the article. Further inquiries can be directed to the corresponding author.
